# Progress in ecotoxicology, environmental chemistry and ecology

**DOI:** 10.1186/s12302-014-0023-4

**Published:** 2014-08-27

**Authors:** Bernd Sures, Nadine Ruchter, Sonja Zimmermann, Michael Eisinger, Henner Hollert

**Affiliations:** 1 Aquatic Ecology, University of Duisburg-Essen, Universitaetsstr. 5, Essen, 45141 Germany; 2 Centre for Water and Environmental Research, University of Duisburg-Essen, Universitaetsstr. 5, Essen, 45141 Germany; 3 Department of Ecosystem Analysis, Institute for Environmental Research, ABBt - Aachen Biology and Biotechnology, RWTH Aachen University, Worringerweg 1, Aachen, 52074 Germany

## Abstract

This Editorial introduces a series of papers on ‘Progress in ecotoxicology, environmental chemistry and ecology’ and was initiated in the context of the Joint SETAC GLB/GDCh Annual Meeting 2013 at the University of Duisburg-Essen. According to the title of the conference ‘Ecotoxicology in an urban context’ (*Ökotoxikologie im urbanen Raum*), a couple of conference contributions dealing with the occurrence and availability of pollutants relevant for organisms in urban environments will be published as papers in this series. Additional contributions with a focus on current developments in any field of ecotoxicology, environmental chemistry or ecology or which specifically address the importance of multiple stressors are welcome. We cordially invite all colleagues who feel they can contribute to the topic to submit a manuscript to ESEU with reference to this series.

Land use changes are of fundamental importance for ecological and ecotoxicological aspects. Globally, one of the most important developments in this context is the still increasing urbanization. Currently, 54% of the world's population lives in urban areas with a continuing trend of people moving from rural to urban areas [1]. Whilst 30% of the world's population was urban in 1950, 66% of the world's population is expected to live in an urban settlement by 2050 [1]. Apart from fundamental effects on nearly all aspects of everyone's daily life, this global trend also severely affects many ecological and ecotoxicological issues. On the other hand, land use changes may also occur if formerly heavily industrialized regions are affected by structural changes in a way that less industry is necessary and areas are converted to become more natural. For example, the Ruhr metropolitan region has been shaped by structural changes following the decline of the mining and heavy industry that were long symbolic of this area. Such conversion processes are often complicated as many contaminated sites remain after the industrial use has ended. Pollutant levels at these sites represent a serious threat for the health of humans and ecosystems and may impede renaturation processes. Taken together, all measures during transition of landscapes in either direction (urbanization or transition from heavy to lower industrial use) will affect ecological and ecotoxicological aspects.

However, the question arises, what is special about ‘urban ecotoxicology’? Still we are dealing with adverse effects of substances on biota. Apart from the fact that there are some typical urban pollutants such as flame retardants and fungicides (e.g. released by house isolations), traffic-emitted pollutants or contaminated soils from the previous industrial use, among others, the most important aspect is the co-occurrence of many stressors that may interact with toxic effects of pollutants. Accordingly, if organisms are confronted with pollutants and additional stressors simultaneously, the toxicity of a substance might differ markedly. Of course, modulation of toxicological effects by multiple stressors is not only relevant for ecotoxicological aspects in urban areas but for all ecotoxicological studies that contain more than one individual as many external and internal factors may modulate the effects of a substance (Figure 1). Apart from considering obvious effects due to competition or trophic interactions, also less evident or even hidden stressors such as parasites have to be taken into account as they may significantly alter the physiological responses of test organisms which are commonly used as biomarkers [2].

**Figure 1:**
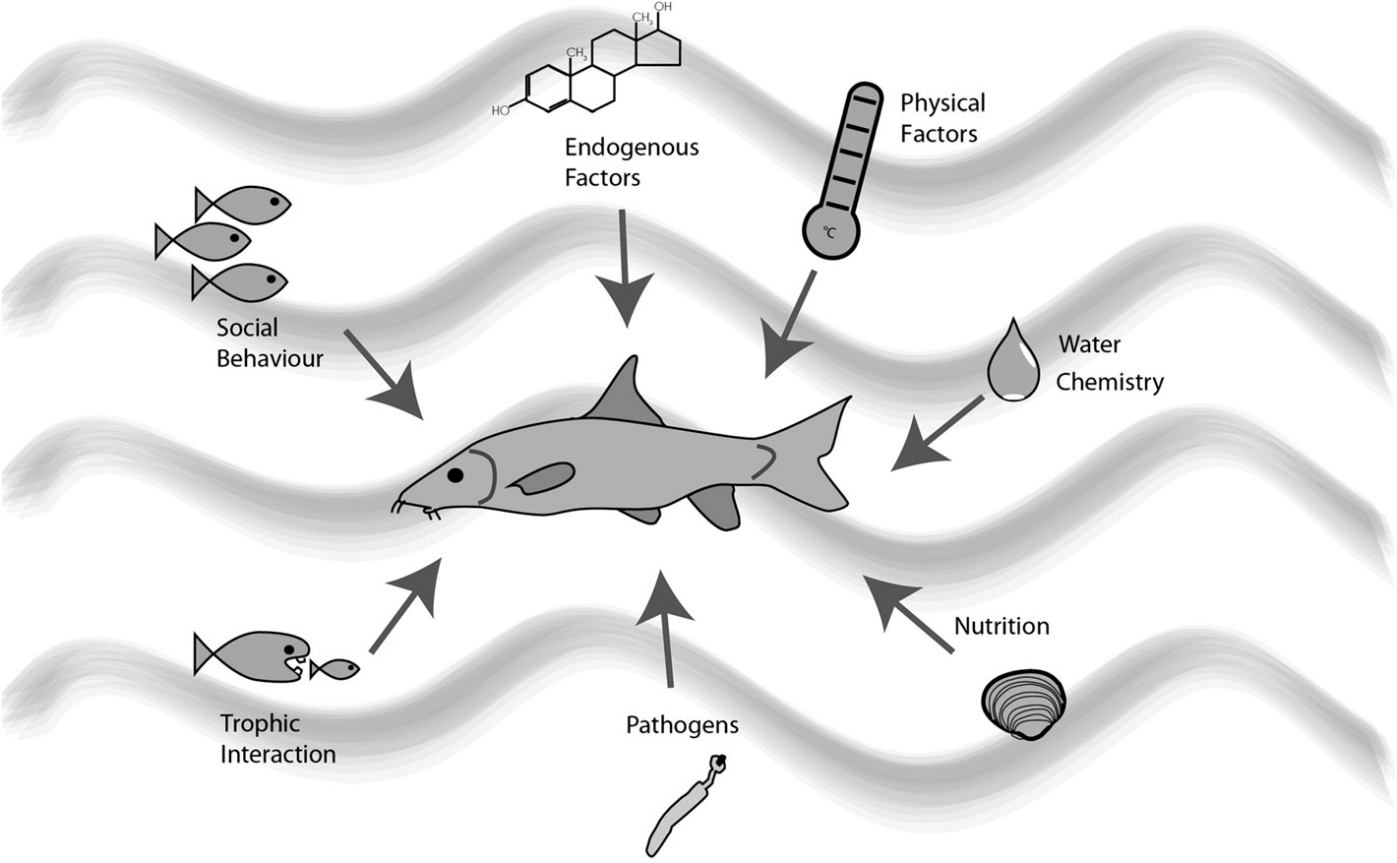
**Physiology and subsequent biomarker responses of organisms are modulated by many internal and external parameters (adapted from Sures et al. [** 3 **]).**

The Joint SETAC GLB/GDCh Annual Meeting 2013 at the University of Duisburg-Essen specifically addressed different fields of interdisciplinary ecotoxicology, such as *Ecotoxicology in an urban context* or *Interaction between parasites and pollutants* in addition to other current issues, such as *Nanoparticles*, *Monitoring of chemicals*, *Biological effect monitoring*, and *Risk assessment*. During the conference, it was decided to initiate the current paper series *Progress in ecotoxicology, environmental chemistry and ecology* and to invite a couple of conference contributions that addressed either of the above-mentioned fields of current ecotoxicological research. Moreover, after the series has started now with some initial contributions from the 2013 conference, the series is open for additional contributions in the form of reviews, research papers or comments. Contributions assigned to this series should present current developments in any field of ecotoxicology, environmental chemistry or ecology, or specifically address the importance of multiple stressors.

We cordially invite all colleagues who feel they can contribute to the topic to submit a manuscript to ESEU with reference to this series.
